# Non-invasive, targeted, and non-viral ultrasound-mediated brain-derived neurotrophic factor plasmid delivery for treatment of autism in a rat model

**DOI:** 10.3389/fnins.2022.986571

**Published:** 2022-09-01

**Authors:** Yuanyuan Shen, Nana Li, Shuneng Sun, Lei Dong, Yongling Wang, Liansheng Chang, Xinyu Zhang, Feng Wang

**Affiliations:** ^1^National-Regional Key Technology Engineering Laboratory for Medical Ultrasound, Health Science Center, School of Biomedical Engineering, Shenzhen University, Shenzhen, China; ^2^Henan Key Laboratory of Medical Tissue Regeneration, School of Basic Medical Sciences, Xinxiang Medical University, Xinxiang, China; ^3^Department of Physiology and Pathophysiology, School of Basic Medical Sciences, Xinxiang Medical University, Xinxiang, China; ^4^Department of Human Anatomy, Histology and Embryology, School of Basic Medical Sciences, Xinxiang Medical University, Xinxiang, China

**Keywords:** focused ultrasound, cationic microbubbles, autism, brain-derived neurotrophic factor, blood-brain barrier

## Abstract

Autism has clinical manifestations such as social interaction disorder, speech and intellectual development disorder, narrow interest range, and stereotyped and repetitive behavior, all of which bring considerable economic and mental burden to society and families, and represent a public health problem requiring urgent attention. Brain-derived neurotrophic factor (BDNF) plays an important role in supporting survival, differentiation, growth, and synapse formation of neurons and participates in the plasticity of nerves. However, it is difficult for BDNF to penetrate the blood-brain barrier (BBB) due to its large molecular weight. Low-frequency focused ultrasound (FUS) combined with microbubbles (MBs) has been demonstrated to be a promising method for opening the BBB non-invasively, transiently, and locally. Here, we studied the therapeutic effect of FUS combined with BDNF plasmid-loaded cationic microbubbles (BDNFp-CMBs) in a rat model of autism. BDNF-CMBs were prepared and the transfection efficiency of FUS combined with BDNF-CMBs was tested *in vitro*. A rat model of autism was established from the juvenile male offspring of Sprague-Dawley (SD) pregnant rats treated with sodium valproate (VPA) solution through intraperitoneal injection. The autism rats were randomized into three groups: the VPA group, which received no treatment, the BDNFp group, which was treated by injection of BDNFp, and the FUS + BDNFp-CMBs group, which was administered FUS combined with BDNFp-CMBs. Age-matched normal rats served as the control group (Con). Following treatment, stereotyped, exploratory, and social–behavioral tests were performed on the animals in each group. The rat brains were then collected for subsequent histological examination, and the changes in synaptic structures in the prefrontal cortex (PFC) were detected under transmission electron microscopy. The results showed that the constructed BDNFp could be loaded onto CMBs with high loading efficiency. The BDNFp-CMBs prepared in this study showed good stability *in vivo*. FUS combined BDNFp-CMBs could effectively and non-invasively open the BBB of rats. The stereotyped, exploratory, and social behaviors of the FUS + BDNFp-CMBs group were significantly improved. Compared to the VPA group, the abnormality of neuronal morphology and number in the PFC of the FUS + BDNFp-CMBs was alleviated to a certain extent and was accompanied by restoration of the damaged synapses in the encephalic region. Our work demonstrates the positive therapeutic effect of BDNF delivered by FUS non-invasively across the BBB into the PFC in a rat model of autism, offering a potential strategy for treating autism.

## Introduction

Autism is a neurodevelopmental disorder, in which the main clinical symptoms include social and communication disorders, stereotyped behavior, and narrow interests (Volkmar and McPartland, 2014). The disorder mostly presents in infants < 3 years old, with significant sex differences (male-to-female ratio of approximately 4:1) (Goldberg et al., 2003). The prevalence rate of the disorder in European and American countries was 0.58‰ in 1987 and approximately 14.7‰ in 2014. According to the latest research data, the prevalence rate of autism in 8-year-old children in the United States is as high as 1/5 (Makushkin et al., 2019; Maenner et al., 2020). In addition to being unable to integrate into society, patients with autism are often accompanied by intellectual and mental disorders, which affect their ability to live independently, resulting in a heavy economic burden on the individuals’ families and society. Therefore, autism has become a public health problem that needs urgent attention. However, its etiology and pathogenesis are still unclear, which brings great difficulties to the treatment of the disorder (Howes et al., 2018).

The prefrontal cortex (PFC) is involved in human social cognition, social behavior, and decision-making ability. Studies have shown that the social ability of rodents is closely related to their PFC, while the abnormal phosphorylation level of the extracellular signal-regulated kinase (ERK) in the PFC of autism model mice is closely related to social disorder (Faridar et al., 2014; Ko, 2017). Therefore, the PFC may be a target brain region for treating autism.

Brain derived neurotrophic factor (BDNF) is a member of a family of neurotrophins, which participates in various neurophysiological processes and is expressed in almost all brain regions, where it regulates the occurrence of nerves and synapses, and affects the mechanisms of short- and long-term memory and cognition (Chou et al., 2020). Studies have shown that after intraperitoneal injection of sodium valproate (VPA) into rats at 12.5 days of gestation to establish an autism rat model, the expression of BDNF in the cerebral cortex of fetal rats can be significantly increased; meanwhile, at 50 days after birth, the expression is significantly reduced, indicating that BDNF is a potential target drug for treating autism (Almeida et al., 2014; Chau et al., 2017). However, because BDNF cannot pass through the blood brain barrier (BBB), intravenous infusion of BDNF cannot effectively reach the brain, and local target injection of BDNF causes damage to brain tissue. Focused ultrasound (FUS) combined with microbubbles can safely and effectively open the BBB (Tosi et al., 2020). In 2001, Hynynen et al. (2001) first confirmed that ultrasound combined with microbubbles can safely and non-invasively open the BBB of rabbits. Since then, ultrasound combined with microbubbles has been widely used in nano drug delivery systems and trans-BBB transport of genes and other small molecules. Using ultrasound combined with BDNF-loaded lipid microbubbles to treat stroke rats can safely open the BBB without damaging brain tissue and effectively deliver BDNF into the brain to enhance the concentration of BDNF and repair the damaged white matter. Other studies in a Parkinson’s disease mouse model have shown that after FUS combined with lipid microbubbles to open the BBB, nasal feeding of BDNF could effectively improve the motor control function of the mice (Rodríguez-Frutos et al., 2016; Shen et al., 2017; Ji et al., 2019; Tosi et al., 2020).

In this study, we prepared cationic microbubbles (CMBs) loaded with BDNF plasmid (BDNFp) to explore their therapeutic effect with FUS to open the rat BBB and deliver the BDNF gene into the brain the PFC in the autism rat model; the aim of which was to provide new ideas and methods for the clinical treatment of patients with autism.

## Materials and methods

### Preparation of cationic microbubbles

DSPC (Lipoid, Ludwigshafen, Germany), DSPE-PEG2000 (Lipoid, Ludwigshafen, Germany), and PEI600 (Sigma, United States) were placed proportionally (mole ratio 9:0.5:0.5) in a test tube. A thin layer of phospholipid was formed on the tube wall under the action of nitrogen flow (0.1 MPa). The orifice of the test tube was sealed with sealing film and holes were generated with a needle on the top of the film. The tube was placed in a 500-mL suction flask and vacuumed for 2–3 h. Next, 5 mL Tris buffer was added to the test tube; the phosphatide suspension was separated into 2.5-mL vials, with 1 mL per vial after ultrasonic oscillation at 55–60°C for 20 min, before conducting gas exchange. The vials were filled with perfluoropropane.

### Preparation of cationic microbubbles loading brain derived neurotrophic factor plasmid-cationic microbubbles

Because CMBs are positively charged and BDNFp (pHBLV-CMV-ZsGreen-BDNF, Hanbio Biotechnology Co., Ltd., Shanghai) is negatively charged, BDNFp can be adsorbed on the surface of CMBs to form BDNFp-CMBs. To directly observe the adsorption of BDNFp on the surface of CMBs, we first prepared fluorescent-labeled CMBs; that is, the DSPE-PEG2000 molar ratio was replaced by DSPE-PEG2000-FITC (Lipoid, Ludwigshafen, Germany), while the other steps were the same as those in 1.2; BDNFp was stained with Propidium Iodide (PI) solution (Beijing Solarbio Technology Co., Ltd., Beijing, China), i.e., 10 μL BDNFp and 10 μL PI working solution were mixed evenly and incubated at room temperature for 5 min in the dark, before adding to 100 μL fluorescent-labeled CMBs and incubating at room temperature for 10 min in the dark before photographing under an upright fluorescence microscope (Nikon, Japan). To observe the adsorption effect between CMBs and BDNFp, CMBs and BDNFp were mixed at the volume ratios of 1:1, 1:2, 1:3, 2:1, and 3:1, incubated at room temperature for 10 min, subjected to 1% agarose gel electrophoresis, and photographed by a gel imaging system (Thermo Fisher Scientific, United States). To observe the adsorption efficiency between the two, 500 μL CMBs were added to 5 μg, 10 μg, 20 μg, or 40 μg of BDNFp, incubated at room temperature for 10 min, and centrifuged at 150 × g for 5 min. The supernatant was removed and its volume was recorded before measuring the concentration of BDNFp with a NanoDrop2000 ultra micro spectrophotometer (Thermo Fisher Scientific, United States). The adsorption efficiency was calculated using the following formula:


(1) $$\mathrm{Adsorption}\;\mathrm{efficiency}=\;\left({\text{M}}_{\text{BDNFp}}-{\text{CS}}_{\text{BDNFp}}\times \text{VS}\right)/{\text{M}}_{\text{BDNFp}}\times \text{100\%}$$

M_BDNFp_: Mass of BDNFp added, CS_*BDNFp*_: BDNFp concentration of supernatant, VS: content of supernatant.

To investigate the stability of BDNFp-CMBs *in vivo*, 1 μL BDNFp and 5 μL CMBs were incubated at room temperature for 10 min to prepare BDNFp-CMBs; then, the skulls of adult SD rats (Guangdong Medical Experimental Animal Center, Guangzhou) (*n* = 6) were removed under continuous anesthesia with 2.5% isoflurane and fixed in the ultra-high resolution small animal ultrasound imaging system (Vevo LAZR, FUJIFILM Visual Sonics, Canada). The skull area was removed and coated with an ultrasonic couplant, and the ultrasonic probe was aimed at the area. Next, 5 μL CMBs and BDNFp-CMBs were diluted with phosphate buffered saline (PBS) to 200 μL. Following tail vein injection, ultrasound was turned on, contrast-mode imaging was performed, and imaging was performed every 30 s. The region of interest (ROI) of the same area in the machine circle was taken to obtain the gray value of each picture.

### Establishment of the autism rat model

Sprague Dawley (SD) rats were used to establish the animal model of autism (Guangdong Medical Experimental Animal Center, Guangzhou). According to the previously reported method for establishing an autism rat model (Schneider and Przewłocki, 2005), we injected VPA (Sigma, United States) at 250 mg/mL into the abdominal cavity of SD female rats on day 12.5 of pregnancy at a dose of 600 mg/kg. The normal control pregnant rats were injected with the same dose of normal saline. The offspring of pregnant rats injected with VPA solution were defined as autism rats (VPA rat), and the offspring of pregnant rats injected with normal saline were defined as normal rats (normal rat). To identify whether the model was successfully established, we detected the growth and development of autistic and normal control rats, weighed the rats at 7, 12, 21, and 70 days, and recorded the eye-opening of rats at 12, 13, 14, 15, and 16 days and scored them. The scoring standard was 0 points for both eyes closed, 1 point for opening one eye, and 2 points for opening both eyes. We then detected the behavioral performance of the autistic and normal control rats. First, we detected the orientation to explore the development of the vestibular sensory nerve and motor function of the rats, placed the rats head down on a 25° smooth inclined plane, and recorded the time spent turning 180°. Then, the motor coordination ability of the rats was tested using a swimming test. First, the constant temperature water bath was heated to 28–29°C, and then the rats were placed in the water for observation for 5–10 s. the positions of their heads, nose, and ears in the water were scored as follows: 0 points for head and nose under the water; 1 point if the nose was below the water surface; 2 points if the head and nose were near the water surface, and the ear was lower than the water surface; 3 points if the head and nose were near the water surface, and the middle part of the ear was above the water surface; and 4 points if the head, nose, and ears were all above the water surface.

### The procedure of blood-brain barrier opening by focused ultrasound combined with brain-derived neurotrophic factor plasmid-cationic microbubbles

The experimental sonication setup is illustrated in Figure 3A. A single-element spherical transducer (center frequency, 971 kHz) was driven by a 50-dB power amplifier (2,100 L, Electronics and Innovation, United States) to generate a FUS beam. The transducer was mounted on an automated stereotaxic apparatus (71,000, RWD Life Science Co., Ltd., Shenzhen, China) for positioning to the target brain region (The PFC, PFC).

**FIGURE 1:**
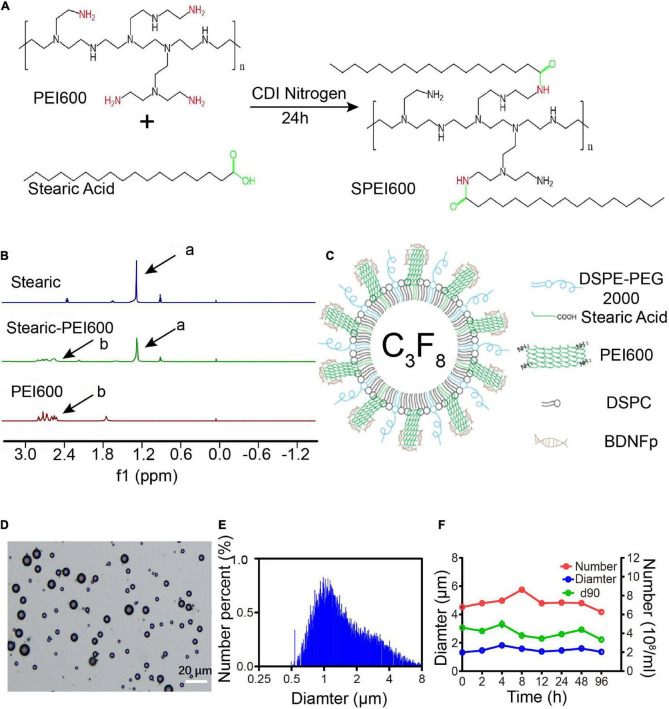
Preparation and characteristics of cationic microbubbles (CMBs). **(A)** SPEI600 synthesis diagram. **(B)** Hydrogen spectra of stearic acid, PEI600, and SPEI600 in CDCl3 (a: - CH2-, b: PEI). **(C)** CMB structure mode diagram. **(D)** Morphological distribution of CMBs. **(E)** CMB particle size distribution. **(F)** Particle size, concentration, and d90 of CMBs at different times.

**FIGURE 2:**
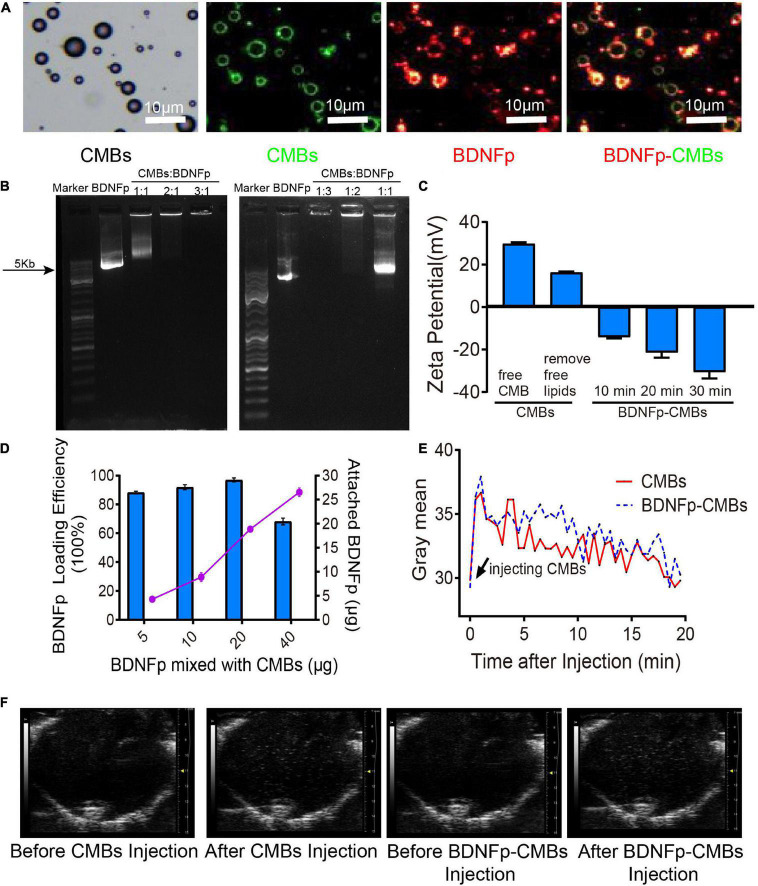
Preparation and characteristics of cationic microbubbles loading brain-derived neurotrophic factor plasmid (BDNFp-CMBs). **(A)** BDNFp adhered to the surface of CMBs under an ordinary optical microscope and *in situ* fluorescence microscope. **(B)** BDNFp-CMBs agarose gel electrophoresis. **(C)** CMBs and BDNFp-CMBs zeta potentials. **(D)** Adsorption efficiency of BDNFp and CMBs. **(E)** The time-intensity curves of CMBs and BDNFp-CMBs following tail vein injections. **(F)** Contrast-mode imaging of two types of microbubbles in the brains of mice before and after tail vein injection of CMBs and BDNFp-CMBs.

**FIGURE 3:**
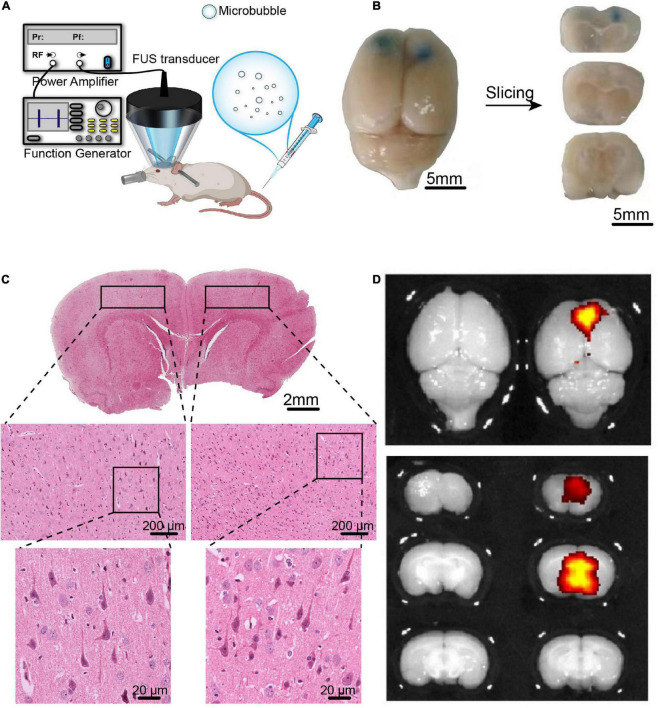
The BBB opening induced by FUS combined with BDNFp-CMBs. **(A)** Illustration of the experimental platform for FUS and BDNFp-CMBs to open the BBB. **(B)** BBB opening area after a single treatment of FUS combined with BDNFp-CMBs revealed by Evans blue extravasation. Bilateral prefrontal cortical regions were sonicated. **(C)** HE staining of the rat brain coronal section after the BBB opening in rats. **(D)** Fluorescence images of EB in the rat brains indicating success BDNF transfection in the sonicated brain region.

Six adult male rats were used to verify the safe opening of the BBB by FUS combined with BDNFp-CMBs. These rats were depilated and anesthetized with 2.5% isoflurane (RWD Life Science Co., Ltd., Shenzhen, China). The body temperature of each rat was maintained through a heating pad. Then, 50 μL BDNFp-CMBs were diluted to 200-μL PBS. At 15 s after the injection of BDNFp-CMBs, the sonication started with a repetition frequency of 1 Hz, a peak negative pressure of 1.48 MPa, a pulse length of 10,000 cycles and a duration of 5 min. Bilateral PFC was sonicated with a 20-min interval time. After sonication, 2 mL 0.2% Evans blue (EB) solution was injected through the tail vein. After 4 h, the rats were perfused with conventional 4% PFA and fixed overnight, and they were imaged with a small animal fluorescence imaging system (IVIS Lumina + XGI-8, Caliper Life Science, United States). The intact brains were embedded in paraffin and sliced into 5-μm sections for hematoxylin and eosin (HE) staining.

### Animal grouping of autistic rats treated with focused ultrasound and brain-derived neurotrophic factor plasmid-cationic microbubbles

Forty 21-day-old male SD rats were randomly divided into four groups as follows: autism rat model group (VPA), direct injection of BDNFp (BDNFp group), ultrasound + BDNFp-CMBs group (FUS + BDNFp-CMBs), and control group (CON). The treatment plan for the BDNFp group was as follows: after the rats were anesthetized by intraperitoneal injection of 2% pentobarbital sodium solution, they were fixed on the brain stereotactic instrument (RWD Life Science Co., Ltd., Shenzhen, China), the head hair was removed, conventional local disinfection was performed, the scalp was cut open to expose the anterior fontanelle and the surrounding area, and conventional wound disinfection was performed. After determining the injection coordinates (2.68 mm forward; 1 mm right; 0.8 mm down), with the anterior fontanelle as the origin, 5 μg BDNFp was injected into the PFC with stereotactic brain positioning, the wound was closed with bone wax, the scalp was sutured, disinfected with antibiotic ointment. The treatment cycle applied for both the BDNFp group and the FUS-BDNFp-CMBs group was once a week for 4 weeks. The treatment plan of the FUS + BDNFp-CMBs group was as follows: anesthesia, fixation, and depilation were performed as outlined above, followed by tail vein injection containing 5 μg BDNFp bound to BDNFp-CMBs. The ultrasonic transducer was aimed at the PFC of the rat. After the scalp was coated with the coupling gel, the ultrasound was turned on. The parameters were as follows: center frequency, 971 kHz; repetition frequency, 1 Hz; peak-negative sound pressure, 1.48 MPa; pulse length, 10,000 cycles; and duration, 5 min. The treatment period was once a week and lasted for 4 weeks.

### Behavioral evaluation of autistic rats treated with focused ultrasound and brain-derived neurotrophic factor plasmid-cationic microbubbles

To evaluate the therapeutic effect, we tested the behavioral performance of the rats in each group. First, we detected the stereotyped behavior of the 69-day-old rats. To this end, the rats were placed in a cage alone and their activity performance within 1 h was recorded with a camera. Two raters watched the video and scored the rats according to the method designed by Costall and Naylor (1973). The scoring standard is 0 points for no stereotyped behavior; 1 point for suspected stereotyped behavior; 2 points for stereotyped behavior; 3 points for strong stereotyped behavior; and 4 points for strong stereotyped behavior and long duration. The average score of the two raters was taken for statistical processing.

Secondly, we detected the exploratory behavior of 71-day-old rats. According to the detection method established by Boissier and Simon (1962), we constructed a rectangular open box with a size of 66 cm × 57 cm × 40 cm, with three small holes in the long walls of 40 and 66 cm, and two small holes in the short wall of 57 cm. The times at which the rat stood upright and inserted its nose into the hole within 5 min were recorded at 10 a.m.

Finally, we detected the social behavior of 75-day-old rats. According to the detection method established by Schneider and Przewłocki (2005) we constructed a self-made detection cage composed of acrylic plate materials, with a size of 43 cm × 28 cm × 15 cm. Seven days before the experiment, the rats to be tested were raised separately and the normal rats were raised in groups as stimulating rats. Two days before the experiment, the rats to be tested and stimulated were placed in the cage separately for 5 min each time to allow them to adapt to the new environment. On the day of the experiment, one rat to be tested and one stimulated rat were placed in the cage for 10 min, and three indicators were recorded, including latency of social behavior, contact times, and average duration.

### Pathological analysis of autistic rats treated with focused ultrasound and brain-derived neurotrophic factor plasmid-cationic microbubbles

After the behavioral tests, the rats were perfused and fixed, and the brains were extracted. After routine paraffin embedding, the coronal sections were stained with Nissl, and the three visual fields of the pyramidal cell layer in the brain PFC were photographed under an ordinary optical microscope to observe the morphology of neurons and count the number of neurons.

To further explore the ultrastructural changes in the PFC of autistic rats treated with FUS and BDNFp-CMBs, rat brains were fixed by perfusion using 2.5% paraformaldehyde and 1.5% glutaraldehyde in 0.1 M phosphate buffer (pH 7.2). Sections measuring approximately 0.5 mm^3^ from the PFC of autistic rats were fixed for 2 h in the same fixative and then treated for electron microscopic observation. Synaptic damage is mainly manifested by blurred synaptic space, indistinguishable presynaptic and postsynaptic membranes, thickening of postsynaptic density (PSD). and reduction of Synaptic vesicles (SVs). To assess the extent of damaged synapses, the ratio of the number of damaged synapses to the total number of synapses was evaluated. Four electron micrographs (8,000×) were randomly selected for each animal in each group. The total number of synapses with clear structure and the number of damaged synapses in each picture were counted, and the synaptic damage rate = the number of damaged synapses/the total number of synapses × 100%. To evaluate the number of synaptic vesicles at nerve endings, the number of 10 SVs at nerve endings per animal was calculated.

### Statistical analysis

GraphPad Prism™ 7.0 (GraphPad Software Inc., San Diego, CA, United States) software was used for statistical analysis of the data, and the measurement data were calculated using the mean ± standard deviation. For the scoring data, non-parametric Kruskal-Wallis tests with Dunn’s multiple comparisons were used. For the other data, one-way ANOVA with Tukey’s multiple comparisons were used.

## Results

### Characteristics of cationic microbubbles

To apply a positive charge to the CMB surface, we coupled stearic acid to SPEI600 under the catalysis of CDI to prepare SPEI600. The synthesis diagram is shown in Figure 1A (Wang et al., 2020). As shown in Figure 1B, HNMR analysis confirmed that the two were successfully coupled to form SPEI600.

SPEI600, DSPC, and DSPE-PEG2000 were mixed in a certain proportion, and CMBs were prepared by a mechanical oscillation method. The structure is shown in Figure 1C. Under an ordinary optical microscope, the CMBs were spherical and scattered with different particle sizes, mostly in the range of 10 μm or less (Figure 1D). As shown in Figures 1E,F, the particle counter test results show that the average particle size of the newly prepared CMBs (0 h) was 1.33 ± 0.24 μm. The average concentration was 4.79 ± 0.35 × 10^8^/mL, and the average d90, below which 90% of CMBs fall, was 3.07 ± 0.18 μm. At 96 h after preparation, the average particle size of CMBs increased to 1.36 ± 0.16 μm, the average concentration decreased from 4.79 ± 0.35 × 10^8^/mL to 4.26 ± 0.13 × 10^8^/mL, and the average d90 decreased from 3.07 ± 0.18 μm to 2.23 ± 0.26 μm. As shown in Figure 2C, the zeta potentiometer test results showed that the surface potential of CMBs was 29.8 MV, and the potential dropped to 16.3 MV after centrifugation to remove the free PEI600. This indicates that CMBs have good characteristics, including appropriate particle size, high concentration, good stability, and positive surface charge.

### Characteristics of brain-derived neurotrophic factor plasmid-cationic microbubbles

Using the principle of positive and negative attraction, we mixed and incubated CMBs and BDNFp to prepare BDNFp-CMBs. The structure is shown in Figure 2C.

To confirm whether BDNFp was adsorbed on the surface of CMBs to form BDNFp-CMBs, CMBs with FITC fluorescence (green) and BDNFp with PI fluorescence (red) were prepared. After 10 min of mixed incubation, the white field and fluorescence photography under the fluorescence microscope is shown in Figure 2A. The layer of red fluorescence on the surface of FITC-labeled CMBs indicates that BDNFp is adsorbed to the surface of the CMBs. The results of agarose gel electrophoresis are shown in Figure 2B. When the content of BDNFp was constant, with the increase in CMBs content, the adsorption of BDNFp by CMBs was increased, resulting in less free BDNFp and shallower and shorter gene bands. When CMBs: BDNFp equals 3:1, the bands almost disappear. In contrast, when the content of CMBs was constant, with the increase of BDNFp content, the free BDNFp was increased, and the gene bands became deeper and longer. These results show that with the increase in CMB content, the adsorption capacity of BDNFp is stronger, with a dose-dependent adsorption capacity. Zeta potentiometers detected the potential of BDNFp CMBs formed after 10 min, 20 min, and 30 min of mixed incubation. The results are shown in Figure 2C. The zeta potentials were −14.1, −21.4, and −30.6 MV, respectively. With the extension of incubation time, increased BDNFp was adsorbed on the surface of CMBs, indicating that the adsorption capacity of CMBs to BDNFp was time dependent. Finally, we quantitatively detected the concentration of free BDNFp with an ultra-micro spectrophotometer and calculated the content of BDNFp. With 5, 10, 20, and 40 μg of BDNFp added in the CMBs solution, the adsorption efficiencies of the CMBs were 88.28, 91.9, 96.9, and 68.2%, respectively, as shown in Figure 2D. In conclusion, CMBs have a strong ability to adsorb BDNFp.

BDNFp adsorbed on the surface of CMBs constitutes CMBs carrying the BDNF gene, namely, BDNFp-CMBs. To explore its stability in the animal brain, CMBs and BDNFp-CMBs were separately injected into the tail vein of rats and then imaged with the ultra-high resolution small animal ultrasound imaging system, as shown in Figures 2E,F. When CMBs or BDNFp-CMBs were injected into mice, the gray value of the brain first increased significantly, and then slowly decreased to the initial value, with a half-life in the brain of approximately 10 min. The results showed that CMB and BDNFp CMBs had good stability in the brain.

### The blood-brain barrier opening induced by focused ultrasound combined with brain-derived neurotrophic factor plasmid-cationic microbubbles

Studies have shown that ultrasound combined with lipid microbubbles can non-invasively open the BBB to deliver BDNF into the brain and increase its concentration. However, combining FUS with BDNFp-CMBs to open the BBB has not yet been studied. Therefore, we first assessed the safety of this technology to open the BBB. As shown in Figure 3B, the PFC of rats sonicated by ultrasound was stained blue by EB, indicating that the BBB was opened in this region. The results of HE staining are shown in Figure 3C. In the fluorescence image, the open area has strong fluorescence, while the area not irradiated by ultrasound has no fluorescence (Figure 3D). There was no red blood cell extravasation and tissue damage, indicating the parameters we used are safe. The results showed that CMBs could safely and effectively open the blood-brain barrier (BBB) in rats.

### Evaluation of the established autism rat model

**FIGURE 4:**
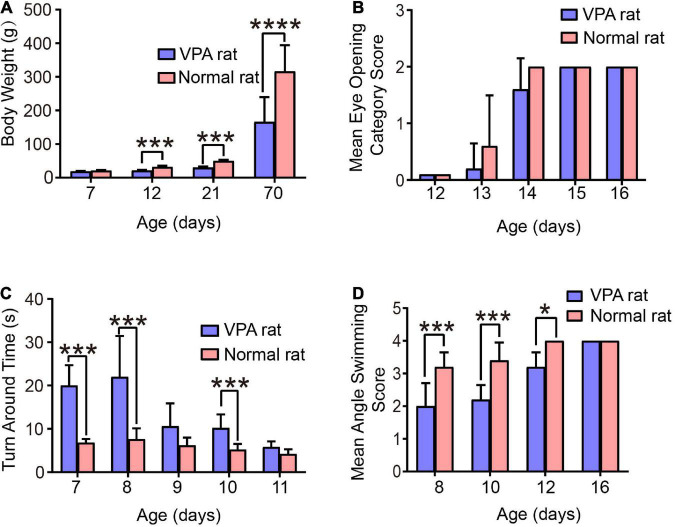
Behavioral evaluation of the VPA-induced autism rats. **(A)** Body weight of rats. **(B)** Eye-opening score of rats. **(C)** Time taken for rats to turn 180°. **(D)** Head position score of rats during swimming. *p < 0.05, ***p < 0.001, ****p < 0.0001.

To evaluate the therapeutic effect of FUS combined with BDNFp-CMBs on autistic rats, we first established an autistic rat model by chemical induction. Then, the success of the model was evaluated by the behavioral performance of the rats. As shown in Figure 4A, compared to normal control rats, the increasing speed of the weight of the autistic rats is significantly slower at the age of 12, 21, and 70 days, especially at the age of 70 days, at which point, the weight of autistic rats was 166.2 ± 73.64 g, while that of normal control rats was 316.4 ± 77.88 g.

The test results of eye-opening are shown in Figure 4B. Compared to normal control rats, the eye-opening scores of autistic rats at the age of 13 and 14 days were significantly lower (*p* < 0.05). At the age of 13 days, the scores of autistic and normal control rats were 0.2 ± 0.45 and 0.26 ± 0.89, respectively; at the age of 14 days, the scores were 1.6 ± 0.55 and 2.0 ± 0.0, respectively; and at the age of 15 and 16 days, the eyes of both groups were completely opened. These results suggest that autistic rats have delayed growth and development.

The orientation tendency of the rats was tested and the results are shown in Figure 4C. Compared to normal control rats, the time taken by autistic rats to turn 180° on a smooth inclined plane at the age of 7, 8, and 10 days was significantly longer, with the most obvious difference observed at 8 days. The time taken by autistic and normal control rats to turn 180° on a smooth inclined plane was 22.0 ± 9.43 s and 7.6 ± 2.51 s, respectively, although the difference was not significant at the age of 9 and 11 days, However, autistic rats tended to take a longer time to turn, indicating that the development of vestibular sensory nerve and motor function in autistic rats is delayed.

Finally, we performed a swimming test on the rats and scored the positions of their heads, noses, and ears. The results are shown in Figure 4D. Compared to normal control rats, the scores of autistic rats at the ages of 8, 10, and 12 days were significantly lower. Among them, the scores of autistic and normal control rats at the age of 10 days were 2.2 ± 0.45 and 3.4 ± 0.55, respectively, and the score at the age of 16 days was 4, indicating that juvenile autistic rats had poor swimming ability. However, with the aging of the rats, their swimming ability improved and returned to normal at the age of 16 days.

### Behavioral evaluation of autistic rats treated with focused ultrasound combined with brain-derived neurotrophic factor plasmid-cationic microbubbles

In the present study, the stereotyped behavior, exploratory behavior, and social behavior were assessed after the autistic rats treated by BDNFp only or FUS + BDNFp-CMBs treatment.

**FIGURE 5:**
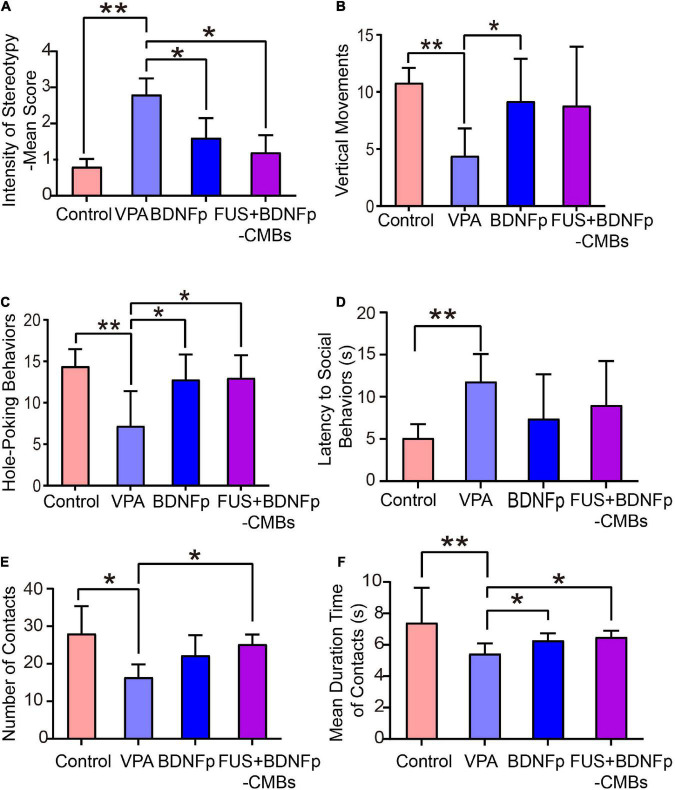
Behavioral evaluation of autistic rats treated with FUS combined with BDNFp-CMBs. **(A)** The stereotyped behavior scores of the rats in the four groups. **(B)** The standing times of rats in the four groups. **(C)** The times of “nose contact hole exploration” of the rats in the four groups. **(D)** The social behavior latency of the rats in the four groups. **(E)** The contact times of the rats in the four groups. **(F)** Contact durations of the rats in the four groups. Con, Control group; VPA, Autism rat model group; BDNFp, Direct injection of the BDNFp group. FUS + BDNFp-CMBs: FUS combined with the BDNFp-CMBs group. The data in **(A–C,E)** were analyzed using non-parametric Kruskal-Wallis tests with Dunn’s multiple comparisons. The data in **(D,F)** were analyzed using one-way ANOVA with Tukey’s multiple comparisons. *p < 0.05, **p < 0.01.

The results of the rat stereotype scores are shown in Figure 5A. The scores of the VPA group, BDNFp group, and FUS + BDNFp-CMBs group were 2.8 ± 0.45, 1.8 ± 0.84, and 1.2 ± 0.45, respectively. Compared to autistic rats (VPA group), the scores of the BDNFp group and FUS + BDNFp-CMBs group decreased significantly, while those of the FUS + BDNFp-CMBs group decreased even further (^***^p < 0.001).

The exploratory behavior was revealed by two indexes: vertical movements and hole-poking times (Figures 5B,C). As shown in Figure 5B, the upright times of the normal control rats (control group), VPA group, BDNFp group, and FUS + BDNFp-CMBs group were 10.8 ± 0.58, 4.4 ± 1.08, 9.2 ± 1.66, and 8.8 ± 2.31, respectively. Compared to the control group, the times of standing upright in the VPA group was significantly reduced. After treatment, compared to the VPA group, the number of times of standing upright of the BDNFp group was significantly increased, and the FUS + BDNFp-CMBs group showed an increasing trend (*p* = 0.1227). However, there was no significant difference between the BDNFp and FUS + BDNFp-CMBs groups. As shown in Figure 5C, the frequencies of hole-poking of the rats in these four groups were 14.4 ± 0.93, 7.2 ± 1.89, 12.8 ± 1.36, and 13 ± 1.23, respectively. Compared to the control group, the frequency of hole-poking in the VPA group was significantly reduced. After treatment, compared to the VPA group, the frequency of hole-poking in the FUS + BDNFp-CMBs group was significantly increased. The results showed that the exploratory behavior ability of autistic rats decreased and improved after BDNFp injection or FUS + BDNFp-CMBs treatment. In the BDNFp group, BDNFp was injected directly into the rat brain, which was an invasive way. However, FUS + BDNFp-CMBs was a non-invasive approach to deliver the BDNFp to the brain. Thus, the results in Figure 5 showing that there was no significant difference between these two groups exactly reflect the fact that FUS + BDNFp-CMBs treatments could achieve comparable outcomes with the direct injection of BDNFp.

As shown in Figures 5D, the latency of social behavior in the VPA group, BDNFp group, and FUS + BDNFp-CMBs group was 11.8 ± 1.46 s, 7.4 ± 2.36 s, and 9.0 ± 2.35 s, respectively. Compared to the VPA group, the latency of social behavior in the BDNFp and FUS + BDNFp-CMBs groups was shorter, but not significantly. Compared to the VPA group, the number of contacts of the FUS + BDNFp-CMBs group increased significantly (*P* < 0.05), while that of the BDNFp group did not (Figure 5E). The contact duration between the three groups and the stimulated rats was 5.43 ± 0.29 s, 6.28 ± 0.2 s, and 6.48 ± 0.19 s, respectively, which was significantly longer than that of the VPA group (*P* < 0.05). The results showed that compared to normal rats, the autistic and stimulated rats had communication disorders to some extent, which were improved after the treatment of FUS combined with BDNFp-CMBs.

### Histological observation of autistic rats treated with focused ultrasound combined with brain-derived neurotrophic factor plasmid-cationic microbubbles

**FIGURE 6:**
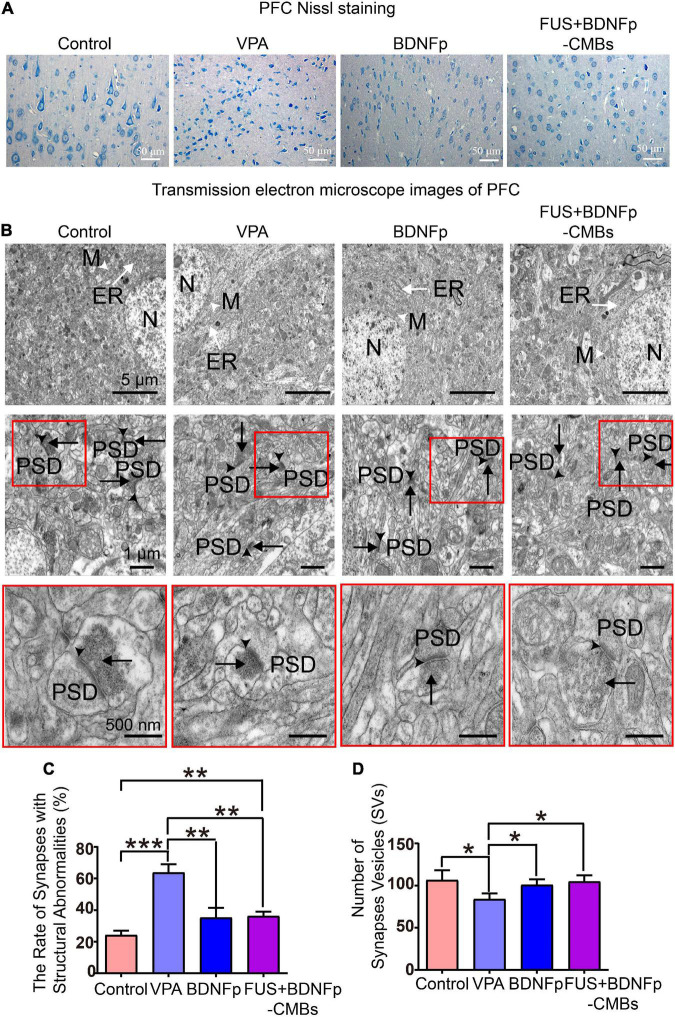
Pathological observation of autistic rats treated with FUS combined with BDNFp-CMBs. **(A)** Ordinary light microscopy of PFC Nissl staining of rats in each group (scale: 50 μm). **(B)** Transmission electron microscopy of the PFC of rats in each group (White tailless arrow: M, mitochondria. White long-tail arrow: ER, endoplasmic reticulum. Black tailless arrow: SC, synaptic cleft. Black long-tail arrow: SVs, Synaptic vesicles. PSD: postsynaptic density). **(C)** The ratio of the number of damaged synapses in the PFC to the total number of synapses in each group. **(D)** The number of SVs in the PFC of rats in each group. Con, Control group; VPA, Autism rat model group; BDNFp, Direct injection of the BDNFp group; FUS + BDNFp-CMBs, FUS combined with the BDNFp-CMBs group, *P < 0.05, **P < 0.01, ***P < 0.001.

The Nissl staining results of the coronal slices of rat brain are shown in Figure 6A. In the control group, the neuronal cell bodies in the PFC were in normal morphology. In the VPA group, the neuronal cell bodies in the PFC were significantly smaller and the nuclei were pyknotic, indicating neuronal damage in this brain region in autistic rats (Abhishek et al., 2022). After treatment, some neurons in the PFC of the BDNFp group recovered to a normal shape, but there were still many damaged neurons; in the FUS + BDNFp-CMBs group, most of the neurons in the PFC returned to normal, both in terms of cell body size and nuclear size.

The results of transmission electron microscopy are shown in Figure 6B. In the control group, the ultrastructure of neurons in the PFC was normal, the boundaries and nuclei of neurons were clear, and the endoplasmic reticulum (ER) and mitochondria (M) were visible in the cytoplasm. There are many synapses around neurons, with a clear structure, clear presynaptic and postsynaptic membranes and synaptic spaces, and obvious staining of PSD. SVs were densely distributed in the presynaptic components, and many vesicles were in direct contact with the presynaptic membrane. Compared to the control group, the VPA group has many blurred synaptic spaces, difficult to distinguish presynaptic and postsynaptic membranes, thickened PSD, and reduced SVs, all of which indicate damage to the synapses formed by neurons in the PFC of autistic rats (Gąssowska-Dobrowolska et al., 2020). Compared to the VPA group, the damaged synapses in the BDNFp group and FUS + BDNFp-CMBs group were improved to some extent, most of the presynaptic, postsynaptic membranes, and synaptic spaces were clear, and the postsynaptic dense substance (PSD) was thin, although damaged synapses were still observed in some areas. The ratio of the number of damaged synapses in the PFC of rats in each group to the total number of synapses was counted. As shown in Figure 6C, the ratios of the control group, VPA group, BDNFp group, and FUS + BDNFp-CMBs group were 24.25 ± 4.66%, 63.75 ± 9.04%, 35.25 ± 10.76%, and 36.25 ± 4.71%, respectively. Compared to the control group, the ratio of the VPA group was significantly higher, while compared to the VPA group, the ratios of the BDNFp group and FUS + BDNFp-CMBs group were significantly lower; however, there was no significant difference between the BDNFp group and the FUS + BDNFp-CMBs group. The number of SVs in the PFC of rats in each group was also counted. As shown in Figure 6D, the numbers of SVs in the control group, VPA group, BDNFp group, and FUS + BDNFp-CMBs group were 106.70 ± 49.83, 84.05 ± 29.19, 100.90 ± 28.38, and 104.80 ± 32.33, respectively. Compared to the control group, the number of SVs in the VPA group was significantly reduced. Compared to the VPA group, the number of SVs in the BDNFp group and FUS + BDNFp-CMBs group was significantly increased, but there was no significant difference between the BDNFp group and FUS + BDNFp-CMBs group. In conclusion, compared to normal rats, the synaptic structure of the PFC was destroyed in autistic rats. The synaptic structural abnormalities were improved after direct injection of BDNFp and FUS combined with BDNFp-CMBs into the PFC.

## Discussion

In this study, we established a recognized autism rat model by injecting sodium valproate (VPA) solution into the abdominal cavity of 12.5-day pregnant rats as reported previously (Schneider and Przewłocki, 2005). To prove the success of the modeling, we tested the growth and development of rats. The results showed that compared to the control rats, the VPA rats had slow weight growth and delayed eye-opening in the early stage of development, indicating delayed growth and development. Then, a series of behavioral performances were tested. The results showed that in the early development, VPA rats took significantly longer to turn 180° on a smooth inclined plane than control rats. When swimming in a constant temperature water bath, the position scores of the rat heads, noses, and ears were significantly lower, but all returned to normal before adulthood, indicating that their motor function development was delayed. In adulthood, compared to control rats, VPA rats have obvious stereotyped behavior, and the ability to explore upright and “nose touch hole” was significantly reduced. Moreover, the incubation period of social behavior was significantly prolonged, the number of contacts with stimulation rats was significantly reduced, and the contact duration was significantly shortened, indicating defective social communication ability of VPA rats. The above results are highly consistent with the previous research results (Schneider and Przewłocki, 2005), indicating that the autism rat model was successfully established.

BDNF plays an important role in treating autism, but BDNF cannot pass through the BBB (Tosi et al., 2020); thus, intravenous infusion of BDNF cannot effectively reach PFC neurons to exert biological effects. Local target injection of BDNF protein in the brain causes damage to brain tissue, affecting its clinical promotion. Studies have shown that ultrasound combined with microbubbles can safely and effectively open the BBB (Shen et al., 2017). Indeed, it has been previously found that ultrasound combined BDNF loaded lipid microbubble technology can safely open the BBB without damaging brain tissue in rats with stroke and can effectively deliver BDNF into the brain to enhance its concentration (Rodríguez-Frutos et al., 2016). The surface of traditional lipid microbubbles contains weak negative charge or no charge, known as neutral microbubbles, and has a limited ability to bind to various molecular compounds (Nomikou and McHale, 2010; Nomikou et al., 2012). Because nucleic acid molecules are negatively charged, if the surface of microbubbles is positively charged (such as CMBs), the two can be effectively combined, which is conducive to carrying nucleic acid molecules. It has been reported that the combination of FUS and glial cell-derived nerve growth factor (GDNF)-loaded CMBs can be used to treat rats with Parkinson’s disease. Compared to neutral microbubbles, the combination of CMBs and GDNF was stronger, which can improve the transfection efficiency of GDNF and has a neuroprotective effect on Parkinson’s rats, successfully blocking the disorder progression and restoring behavioral function (Fan et al., 2016). Therefore, under ultrasonic irradiation, CMBs could not only open the BBB but also effectively enhance the gene loading ability and gather more drugs to the target area to achieve a better therapeutic effect.

Our prepared CMBs have good characteristics, including appropriate particle size, high concentration, positive surface charge, and are relatively stable *in vivo* and *in vitro*. Because BDNFp is negatively charged, CMBs and BDNFp can combine to form a BDNFp-CMBs complex after mixed incubation. The fluorescence results showed that BDNFp was adsorbed on the surface of CMBs. When the content of BDNFp was constant, increasing BDNFp was adsorbed by CMBs with increasing CMB content. When the content of CMBs was constant, free BDNFp increased with the increase in BDNFp content. With the prolongation of incubation time, more BDNFp was adsorbed on the surface of CMBs, indicating that the adsorption capacity of CMBs for BDNFp was both dose- and time-dependent. Additionally, the ultra-micro spectrophotometer results showed that the adsorption efficiency of CMBs for BDNFp was between 68.2 and 96.9%. In conclusion, CMBs have a strong adsorption capacity for BDNFp. After BDNFp-CMBs were injected into the tail veins of mice, the results of an ultra-high resolution small animal ultrasound imaging system showed that the half-life of BDNFp-CMBs in the brain was approximately 10 min, indicating that BDNFp-CMBs have good stability in the brain. In conclusion, our BDNFp-CMBs have good characteristics and stability *in vivo*, which provides an important experimental basis for the later opening of the BBB and the delivery of BDNFp into the brain. We used the FUS + BDNFp-CMBs technique along with EB staining, small animal fluorescence imaging, and HE staining to explore the opening of the BBB in the PFC of experimental animals. The results showed that the PFC of mice had strong fluorescence, while the other brain regions that were unirradiated by ultrasound had no fluorescence, indicating that BDNFp-CMBs had passed through the BBB into the PFC and that BDNFp had also been transfected into nerve cells in this region. The PFC of ultrasound irradiated rats was stained blue by EB, indicating that the BBB was opened in this region. After HE staining, the tissue structure of this brain area was regular and orderly without obvious bleeding, demonstrating that the BBB was opened and the tissue was not damaged. Therefore, FUS + BDNFp-CMBs can open the rat BBB non-invasively, safely, and in a targeted manner.

To explore the therapeutic effect of FUS + BDNFp-CMBs in autistic rats, we first examined the behavior of rats in the control group, VPA group, BDNFp group, and FUS + BDNFp-CMBs group. The results showed that compared to the VPA group, the stereotyped behavior scores of the BDNFp group and FUS + BDNFp-CMBs group were significantly lower, indicating that the stereotyped behavior of autistic rats was improved after direct injection of BDNFp and FUS + BDNFp-CMBs into the PFC of rats. Compared to the VPA group, the number of upright times in the BDNFp group was significantly increased, with an increasing trend in the FUS + BDNFp-CMBs group, but no significant difference between the BDNFp and FUS + BDNFp-CMBs groups. The number of “nose to explore holes” in the BDNFp and FUS + BDNFp-CMBs group increased significantly, but there was no significant difference between the BDNFp and FUS + BDNFp-CMBs groups. These findings demonstrate that after direct injection of BDNFp and FUS + BDNFp-CMBs into the rat PFC, the low upright, “nose to explore holes,” and other exploration behavior was improved in the autistic rats, but the effects of the two treatment methods were similar. Compared to the VPA group, the latency of social behavior in the BDNFp and FUS + BDNFp-CMBs groups was shorter, but not significant. The times of contact with stimulated rats in the FUS + BDNFp-CMBs group increased significantly, while the duration of contact with stimulated rats was significantly prolonged. These findings indicate that the communication disorder in autistic rats improves to a certain extent after treatment with FUS + BDNFp-CMBs.

To further explore the therapeutic effect of FUS + BDNFp-CMBs on autistic rats, we used Nissl staining to observe the morphology of PFC neurons in the four groups of rats. The results showed that the cell bodies of neurons in the PFC in the VPA group became smaller and the nuclei became pyknotic, highlighting neuron damage in this brain region of autistic rats, which was highly consistent with the previous research results (Abhishek et al., 2022). After treatment, a small part of the damaged neurons in the PFC of the BDNFp group recovered, and in the FUS + BDNFp-CMBs group, the damaged neurons in the PFC recovered completely.

Studies have shown that synapses may be an important site for the occurrence of autism. The specific synaptic protein deficiency may lead to abnormal neurotransmission of excitatory and inhibitory synapses, destroy the balance between excitatory and inhibitory synapses, and affect neural plasticity, which may be one of the basic causes of autism pathology (Won et al., 2013; Torres et al., 2017). The synaptic ultrastructure in the PFC and hippocampus of autistic rats was abnormal, as evidenced by synaptic swelling, a significant decrease in SVS bulk density, a blurred and thickened synaptic space, and the inability to distinguish presynaptic and postsynaptic membranes. Additionally, in most synapses, PSD was fuzzy and thickened, in addition to atrophy, swelling, or elongation of synaptic mitochondria (Gąssowska-Dobrowolska et al., 2020). Therefore, we observed the ultrastructure of the PFC in four groups using transmission electron microscopy. The results showed that the ultrastructure of the PFC in the control group was normal, the neuronal cell boundary and nucleus were clear, and the ER and mitochondria (M) were visible in the cytoplasm. Moreover, many synapses were observed around the neurons with a clear structure, clear presynaptic, postsynaptic membranes and synaptic spaces, and obvious staining of PSD. SVS are densely distributed in the presynaptic components, and many vesicles are in direct contact with the presynaptic membrane. Compared to the control group, the VPA group had many blurred synaptic spaces, with difficult to distinguish presynaptic and postsynaptic membranes, thickened PSD, and a significant decrease in SVS. Compared to the VPA group, the synaptic pathological changes in the BDNFp and FUS + BDNFp CMB groups were improved to some extent. Most presynaptic, postsynaptic membranes and synaptic spaces were clear, the PSD was thin, and the number of SVs had recovered, but pathological synapses were still observed in some areas. Our results showed that although there were no abnormalities in the PFC of autistic rats, such as synaptic swelling, a significant decrease in the bulk density and atrophy of SVs, and swelling or elongation of mitochondria in synapses, the synaptic structure did have abnormalities, which may lead to impaired synaptic function, resulting in impairment of synaptic plasticity and neurotransmission function. Studies have found that synaptic dysfunction is the fundamental cause and key mechanism of autism and many other neuropsychiatric and cognitive disorders (Guang et al., 2018). Some studies have confirmed that the application of FUS combined with microbubble technology can safely open BBB, deliver drugs or genes into the brain, and effectively treat a variety of brain diseases (Aryal et al., 2014; Meairs, 2015; Timbie et al., 2015), and this method was first used in clinical trials in 2018 to treat brain tumors, Alzheimer’s disease and amyotrophic lateral sclerosis. However, the research on the treatment of autism with this technology has not been reported yet.

## Conclusion

In this study, we employed FUS combined with microbubble technology to safely and non-invasively open the BBB of rats, deliver BDNFp into the brain, treat autistic rats and evaluate the treatment effect through behavioral detection and histological observation. To the best of our knowledge, there have been no previous reports of this type that are innovative and have important clinical application value.

## Data availability statement

The original contributions presented in this study are included in the article/supplementary material, further inquiries can be directed to the corresponding author/s.

## Ethics statement

The animal study was reviewed and approved by Ethics Committee of Shenzhen University.

## Author contributions

YS and SS conceived and performed the experiments, performed the data analysis, and wrote the manuscript. LD and YW conducted the study. NL, LC, and FW revised the manuscript and approved the final version. All authors contributed to the article and approved the submitted version.
